# Growth differentiation factor-15 and incident chronic kidney disease: a population-based cohort study

**DOI:** 10.1186/s12882-021-02558-w

**Published:** 2021-10-27

**Authors:** Xue Bao, Biao Xu, Yan Borné, Marju Orho-Melander, Olle Melander, Jan Nilsson, Anders Christensson, Gunnar Engström

**Affiliations:** 1 Department of Cardiology, Nanjing Drum Tower Hospital, the Affiliated Hospital of Nanjing University Medical School, No. 321 Zhongshan Road, Nanjing, 210008 China; 2 Department of Clinical Sciences, Lund University, CRC 60:13, Jan Waldenströms gata 35, 205 02 Malmö, Sweden

**Keywords:** Growth differentiation factor 15, Chronic kidney disease, Estimated glomerular filtration rate, Competing risk, Cohort study

## Abstract

**Background:**

The relationship between growth differentiation factor 15 (GDF-15) and the development of chronic kidney disease (CKD) is still unclear. We sought to examine whether plasma GDF-15 was related to incident CKD and kidney function decline using a large prospective cohort study.

**Methods:**

4318 participants of the Malmö Diet and Cancer Study-Cardiovascular Cohort were examined in 1991-1994. Incidence of CKD was followed prospectively by linkage with national patient registers. Estimated glomerular filtration rate (eGFR) was available for all participants at baseline, and was re-measured in a subgroup of 2744 subjects after 16.6 ± 1.49 years. Incidence of CKD was examined in relation to GDF-15 using Cox regression analysis. Logistic regression was used to examine the association of GDF-15 with eGFR change and eGFR-based CKD. Models were carefully corrected for potential confounders including baseline eGFR, N-terminal pro-B-type natriuretic peptide, and competing risk from death.

**Results:**

165 patients developed CKD after 19.2 ± 4.04 years of follow-up. The adjusted hazard ratio (95% confidence interval, CI) for CKD in 4th versus 1st quartile of GDF-15 was 2.37 (1.33, 4.24) (*p* for trend < 0.01). Each per 1 standard deviation increase in GDF-15 was associated with a decline in eGFR of − 0.97 mL/min/1.73 m^2^ (95% CI, − 1.49 ~ − 0.45; *p* < 0.001). GDF-15 was also significantly associated eGFR-based CKD in 2713 subjects with baseline eGFR ≥60 mL/min/1.73 m^2^.

**Conclusions:**

GDF-15 predicted incidence of CKD and eGFR decline in the general population, independent of a wide range of potential risk factors and competing risk of death.

## Introduction

Around 9.1% of the global population are suffering from chronic kidney disease (CKD), accounting for 35.8 million disability-adjusted life-years and 1.2 million deaths annually [1]. Treatment of CKD can be costly, in particular for patients with end-stage renal disease (ESRD), which poses a considerable financial burden to families and health systems [2]. Fortunately, CKD is preventable with early detection and timely intervention [3, 4]. Efforts have thus been made to develop efficient screening strategies for CKD. In this regard, biomarkers have drawn increasing attention as they may not only help identify high-risk individuals but also provide insights into mechanism of kidney injury.

CKD is a major risk factor for CVD [1, 5], and vice versa, cardiac dysfunction could also lead to kidney injury [6]. Due to the interdependency of heart and kidney, several studies have explored kidney disease in relation to cardiovascular biomarkers, among which the predictive value of growth differentiation factor 15 (GDF-15) was recognized [7–13]. Most available evidence has focused on patients with existing kidney pathology [9–12]. For instance, GDF-15 was found to predict estimated glomerular filtration rate (eGFR) decline and mortality in type 1 diabetic patients with nephropathy [9], mortality in ESRD [10], eGFR decline and progression to ESRD in CKD [11], and progression to dialysis and mortality in light chain amyloidosis [12]. Cross-sectionally, GDF-15 was negatively associated with eGFR and was higher in the elderly with than without CKD [13]. However, community-based data regarding kidney function decline in relation to GDF-15 are scarce. To our knowledge, there were only two relevant studies. Participants in the study by Carlsson, et al. [8] were limited to elderly people. The authors demonstrated that GDF-15 did not predict decline of eGFR independently of baseline eGFR. In contrast, Ho, et al. [7] reported a positive association of GDF-15 with incident CKD, but some potential confounders (smoking, obesity, C-reactive protein, etc. [14]) were not adjusted for. In addition, incident CKD was identified by calculation of eGFR < 60 mL/min/1.73 m^2^, and no information about clinical diagnoses were included.

Therefore, we aimed to investigate the association of GDF-15 with incident CKD, as obtained from national registers, as well as from eGFR calculation. Analyses were performed in a prospective study with a large general population sample and a long-term follow-up, while taking into account potential baseline confounders and competing risk from death.

## Methods

### The Malmö diet and Cancer cardiovascular (MDC-CV) cohort study

**Fig. 1:**
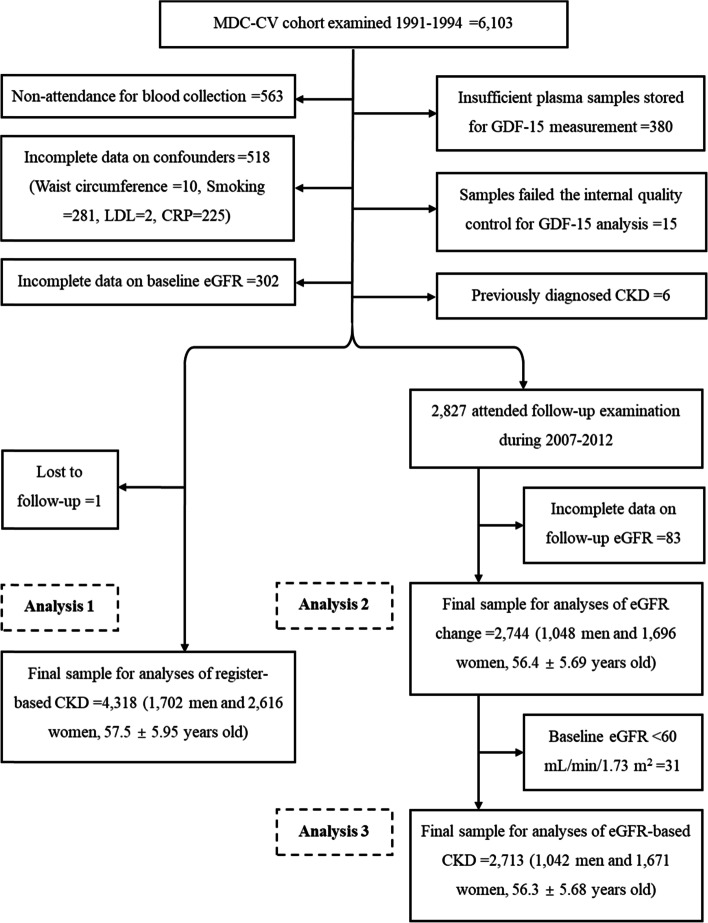
Population flow chart

The MDCS is a large prospective cohort study with participants recruited from Malmö, a city in southern Sweden [15]. During 1991-1994, a random sample of 6103 participants was taken from MDCS to investigate the epidemiology of carotid artery atherosclerosis (MDC-CV cohort study) [14]. Among them, 5540 donated fasting blood samples. We excluded participants with missing baseline data on eGFR (*N* = 302), waist circumference (*N* = 10), smoking (*N* = 281), low density lipoprotein (LDL, N = 2), C-reactive protein (CRP, *N* = 225), or GDF-15 (*N* = 395), or participants with previously diagnosed CKD (*N* = 6) or lost to follow-up (*N* = 1). Therefore, 4318 participants (Fig. 1, analysis 1, mean aged 57.5 ± 5.95 years, male 39.4%) remained for analyses of incident CKD, as detected by national registers of hospital inpatients and outpatients [16].

During 2007-2012, MDC-CV participants who were still alive and living in the Malmö area were invited to a re-examination. A total of 3734 attended, which corresponds to 75.8% of the eligible population [17]. Among the 4318 individuals in this study, 2827 attended re-examination and 2744 had follow-up data available for eGFR. This sub-cohort study was then analyzed for decline in eGFR (Fig. 1, analysis 2). Incident CKD based on eGFR was further analyzed as the outcome in 2713 participants with baseline eGFR ≥60 mL/min/1.73 m^2^ (Fig. 1, analysis 3). Written informed consent was obtained from all included participants. The study conformed to the Declaration of Helsinki and was approved by the ethical committee at Lund University, Lund, Sweden (LU 51/90).

### GDF-15 measurement

Fasting blood samples were collected from the cubital vein and stored at − 80 °C until assay. GDF-15 levels were measured by the SciLifeLab analysis service (Uppsala, Sweden) using Proseek® Multiplex CVD I^96 × 96^ reagent kit where a Proximity Extension Assay technology was applied [14, 18]. Briefly, the assay procedure consisted of three key steps: incubation, extension and detection. Raw Proseek data went through a pre-processing normalization procedure and were set relative to a fixed background level, after which Normalized Protein Expression (log2 scale) values were generated, measured in arbitrary units (AU). High AU values corresponded to a high protein concentration. GDF-15 levels in 987 subjects measured by Proseek assay closely correlated (*r* = 0.89 [L. Lind, unpublished data]) with the values by an electrochemiluminescence immunoassay (Roche Diagnostics, Mannheim, Germany) [14].

### CKD based on the ICD codes from the national register

Information on CKD diagnosis was obtained from the Swedish patient register with nation-wide coverage. Moreover, the Swedish renal registry was searched for any additional cases of CKD [19]. CKD was defined as codes 585-586 according to ICD-9, and N18 and N19 according to ICD-10. All participants without any previous diagnosis of CKD were followed from baseline until the occurrence of a diagnosis of CKD (registry-based CKD), emigration from Sweden, death or December 31st, 2013, whichever came first. Participants were considered to have develop acute kidney injury (AKI) if they developed codes 580, 584 or 590 according to ICD-9, or N00, N10 or N17 according to ICD-10.

The CKD diagnosis in the Swedish patient register has been previously described and validated [20]. Briefly, for validation, CKD diagnoses were evaluated by two experienced specialists in nephrology. Patient records and laboratory data were reviewed and CKD cases were defined following the 2012 KDIGO criteria [21]. Validation showed that 94% of patients had a correct diagnosis of CKD [20].

### CKD based on eGFR, and eGFR decline from baseline to follow-up

eGFR at baseline and follow-up was determined from plasma creatinine and cystatin C using the CKD-Epidemiology Collaboration 2012 eq. [22]. Single measurements of eGFR were assessed at each time point. A cut-off value of 60 mL/min/1.73 m^2^ was used to identify participants with eGFR-based CKD [20]. The difference between these two measurements was defined as eGFR change.

At baseline, creatinine and cystatin C were analyzed with the Jaffé method (Beckman Synchron LX20-4; Beckman-Coulter) and with a particle-enhanced immunonephelometry assay (N Latex Cystatin; Dade Behring, Deerfield, IL), respectively. Since the world calibrator was not introduced until 2010, cystatin C values were not standardized (reference value: 0.53 ~ 0.95 mg/L). During 2007-2012, creatinine was determined in follow-up samples using an enzymatic method (Cobas autoanalyzer; Roche Diagnostics) calibrated by isotope-dilution mass spectrometry-traceable (IDMS) creatinine [23], and cystatin C was analyzed using an automated particle-based immunoassay, adjusted to the international reference preparation ERM-DA 71/IFCC.38 T [24]. Therefore, values of creatinine and cystatin C could not be directly compared between baseline and follow-up.

### Other variables and definitions

Baseline characteristics were obtained from self-administered questionnaire, physical examination, and blood measurements. Data on medication, history of kidney stone, smoking habits and alcohol consumption were collected by questionnaires. Participants were classified into current smokers, former smokers and never smokers. An average daily alcohol consumption > 40 g for males or > 30 g for females was considered as high alcohol consumption. Waist circumference was determined as being midpoint between the end of the 12th rib and the iliac crest. Blood pressure was measured with a mercury-column sphygmomanometer after 10 min of rest while the subject was in a supine position. Participant with a history of coronary event or stroke was considered to have CVD at baseline.

Glucose concentration was measured in fresh whole blood samples after an overnight fasting, following standard procedures at the Department of Clinical Chemistry, University Hospital Malmö. Diabetes was defined as self-reported physician diagnosis of diabetes, use of anti-diabetic drugs or fasting whole blood glucose ≥6.1 mmol/L (corresponding to plasma glucose ≥7.0 mmol/L). LDL concentration was estimated using the Friedewald’s formula. Measurements of biomarkers were conducted later using frozen (− 80 °C) plasma samples. CRP was measured with a Tina-quant® CRP latex assay (Roche Diagnostics, Basel, Switzerland). Methods to measure N-terminal pro-B-type natriuretic peptide (NT-proBNP) levels was the same way as that for GDF-15 [14, 18].

### Statistical analyses

Baseline characteristics are presented for participants divided into quartiles (Q1-Q4) according to GDF-15 concentration, using sex-specific quartile limits. For skewed variables, log-transformation was performed to achieve a normal distribution. Differences across GDF-15 quartiles were examined using analysis of variance for continuous variables and logistic regression analysis for categorized variables.

Cox proportional hazard regression was used to analyze the association between baseline GDF-15 and incident CKD discovered by the national register. Hazard ratios (HRs) and 95% confidential intervals (CIs) were obtained. GDF-15 was treated both as a continuous variable (per standard deviation (SD) change) and as a categorized variable (in quartiles). In multivariate-adjusted models, potential covariates taken into consideration were age, sex, waist circumference, smoking, high alcohol consumption, systolic blood pressure, LDL, CRP, diabetes, CVD, anti-hypertensive drug medication, and baseline eGFR. Since GDF-15 has been frequently considered as a cardiovascular biomarker in recent years, NT-proBNP, a traditional cardiovascular marker was additionally adjusted for in a sensitivity analysis to explore whether the association of GDF-15 with CKD could be mediated by cardiac function. History of kidney stone was also included as a covariate in another sensitivity analysis (*N* = 4309). A restricted cubic spline function was incorporated into the Cox model to test for possible non-linearity, with knots placed at 20, 40, 60 and 80 percentages of GDF-15 concentration. Possible effect modifications were examined by introducing an interaction term between GDF-15 levels and risk factors into the multivariate model one by one. The competing risks of death was accounted for in a sensitivity analysis by the Fine and Gray proportional subdistribution hazards models method. In another sensitivity analysis, the association between GDF-15 and CKD was analyzed while participants with baseline eGFR < 60 mL/min/1.73 m^2^ were excluded. In addition, for participants with follow-up data available for eGFR, multiple linear regression was used to analyze the association between GDF-15 and eGFR change from baseline to the end of the follow-up. A multiple logistic regression analysis was conducted for the association between GDF-15 and eGFR-based CKD. Since CKD can be a consequence of AKI, the associations of GDF-15 with both registry- and eGFR- based CKD were re-estimated after excluding those with incident AKI before the development of CKD.

All analyses were performed using the Statistical Analysis System version 9.3 for Windows (SAS Institute Inc., Cary, NC, USA). A 2-tailed *p* < 0.05 was considered statistically significant.

## Results

### Baseline characteristics

The mean GDF-15 concentration in the cohort was 8.75 ± 0.56 AU and mean eGFR was 89.2 ± 0.56 mL/min/1.73 m^2^. The clinical and biochemical characteristics of the population across GDF-15 quartiles are shown in Table 1. As compared to participants with relatively low GDF-15 concentration, those with higher GDF-15 concentration tended to have decreased eGFR at baseline. An increasing trend was observed for most of the other covariates, except for sex and high alcohol consumption.

**Table 1:** Characteristics of individuals across quartiles (Q1-Q4) of growth differentiation factor-15 (GDF-15)

	GDF15 quartiles				*p* for trend ^a^
	Q1 (n = 1080)	Q2 (*n* = 1079)	Q3 (*n* = 1080)	Q4 (n = 1079)	
GDF-15 (AU)	8.09 ± 0.26	8.56 ± 0.11	8.90 ± 0.14	9.47 ± 0.38	< 0.0001
Age (years)	54.6 ± 5.60	56.6 ± 5.70	58.6 ± 5.50	60.1 ± 5.60	< 0.0001
Sex (male, %)	426 (39.4)	425 (39.4)	426 (39.4)	425 (39.4)	0.99
Waist circumference (cm)	81.6 ± 11.6	82.3 ± 12.3	83.5 ± 12.9	85.3 ± 13.5	< 0.001
Fasting glucose (mmol/L) ^b^	4.80 (4.60-5.20)	4.90 (4.60-5.20)	4.90 (4.60-5.30)	5.00 (4.70-5.40)	< 0.0001
Systolic blood pressure (mmHg)	136.1 ± 16.7	140 ± 18.9	142.4 ± 19.3	144.7 ± 19.3	< 0.0001
Diastolic blood pressure (mmHg)	85.6 ± 8.60	86.6 ± 9.40	86.9 ± 9.50	87.5 ± 9.60	< 0.001
Low-density lipoprotein cholesterol (mmol/L)	4.09 ± 0.94	4.05 ± 0.94	4.25 ± 0.99	4.24 ± 1.03	< 0.001
C-reactive protein (mg/L) ^b^	1.00 (0.50-1.90)	1.10 (0.60-2.30)	1.40 (0.70-2.90)	1.90 (1.00-4.20)	< 0.0001
eGFR (mL/min/1.73 m^2^)	94.3 ± 12.5	90.5 ± 12.2	88.1 ± 12.5	83.8 ± 14.5	< 0.0001
N-terminal pro-B-type natriuretic peptide (AU)	− 0.31 ± 0.92	−0.06 ± 0.94	0.09 ± 0.97	0.26 ± 1.03	< 0.0001
High alcohol consumption (%)	42 (3.89)	37 (3.43)	34 (3.15)	34 (3.15)	0.31
Smoking n (%)					
Never	528 (48.9)	458 (42.4)	420 (38.9)	369 (34.2)	< 0.0001
Former	451 (41.8)	428 (39.7)	410 (38.0)	340 (31.5)	< 0.0001
Current	101 (9.35)	193 (17.9)	250 (23.1)	370 (34.3)	< 0.0001
Anti-hypertensive medication (%)	121 (11.2)	150 (13.9)	186 (17.2)	235 (21.8)	< 0.0001
Diabetes (%)	40 (3.70)	59 (5.47)	81 (7.50)	144 (13.3)	< 0.0001
Cardiovascular disease (%)	9 (0.83)	22 (2.04)	28 (2.59)	43 (3.99)	< 0.0001

*AU* arbitrary units, *eGFR* estimated glomerular filtration rate according to the combined Chronic Kidney Disease Epidemiology Collaboration creatinine and cystatin C equation

^a^ Analysis of variance or logistic regression analysis

^b^ Glucose and C-reactive protein are presented as median (interquartile range in brackets) due to skewed distributions. All the other continuous values are presented as means ± standard deviation, unless otherwise stated

### Incidence of register-based CKD in relation to GDF-15 (analysis 1)

During a mean of 19.2 ± 4.04 years of follow-up, a total of 165 subjects developed CKD. After multivariate adjustment (Table 2, Model 3), an increased GDF-15 level was associated with a higher risk of developing CKD. The HR (highest vs. lowest quartiles of GDF-15) for incident CKD was 2.74 (95% CI, 1.53 ~ 4.89; *p* for trend < 0.001). This value was slightly attenuated after additionally adjusting for baseline eGFR (HR, 2.37; 95% CI, 1.33 ~ 4.24; *p* for trend < 0.01). The adjusted HR for each 1 SD increase in GDF-15 was 1.39 (95% CI, 1.16 ~ 1.65; *p* < 0.001). In the sensitivity analysis when NT-proBNP was additionally added into the model, the association between GDF-15 and CKD hardly changed (HR: 1.35, 95% CI, 1.13 ~ 1.62; *p* < 0.01, per 1 SD increment of GDF-15). However, no statistical significance was observed for NT-proBNP (*p* = 0.10).

**Table 2:** Incident cases of register-based chronic kidney disease in relation to growth differentiation factor-15 (GDF-15)

	Q1	Q2	Q3	Q4	*p* for trend ^a^	Per 1 standard deviation change in GDF-15	*p* ^a^
GDF-15 range male (AU)	6.09-8.47	8.47-8.82	8.82-9.21	9.21-11.2		–	
GDF-15 range female (AU)	6.78-8.35	8.35-8.66	8.66-9.02	9.02-12.3		–	
No. of subjects	1080	1079	1080	1079	–	–	–
Incidence ^b^	16	31	43	75	–	–	–
Incidence (per 1000 person-years)	0.74	1.45	2.08	3.93		–	–
Model 1 ^c d^	Reference	2.08 (1.14, 3.81)	3.15 (1.77, 5.60)	6.58 (3.82, 11.3)	< 0.0001	1.97 (1.75, 2.22)	< 0.0001
Model 2 ^c e^	Reference	1.66 (0.90, 3.04)	1.98 (1.10, 3.56)	3.46 (1.97, 6.10)	< 0.0001	1.81 (1.56, 2.10)	< 0.0001
Model 3 ^c f^	Reference	1.56 (0.85, 2.86)	1.66 (0.91, 3.00)	2.74 (1.53, 4.89)	< 0.001	1.51 (1.27, 1.80)	< 0.0001
Model 4 ^c g^	Reference	1.57 (0.85, 2.88)	1.62 (0.90, 2.92)	2.37 (1.33, 4.24)	< 0.01	1.39 (1.16, 1.65)	< 0.001

*AU* arbitrary units

^a^ Analysis by Cox proportional hazards model

^b^ Defined as 585-586 according to International Classification of Diseases 9, and N18 and N19 according to International Classification of Diseases 10

^c^ Adjusted hazard ratios (95% confidence interval)

^d^ Crude model

^e^ Adjusted for age and sex

^f^ Additionally adjusted for waist circumference, smoking, high alcohol consumption, systolic blood pressure, low-density lipoprotein cholesterol, C-reactive protein level, diabetes, cardiovascular disease, and anti-hypertensive drug medication

^g^ Additionally adjusted for baseline estimated glomerular filtration rate

No obvious evidence of non-linearity in the association between GDF-15 and CKD was detected by restricted cubic spline function (*p* for effect test < 0.0001, *p* for non-linearity test = 0.26). Meanwhile, no interaction between GDF-15 and covariates was found with respect to CKD. During the follow-up period, 952 individuals died from causes other than CKD. When competing risk of death was taken in consideration, the adjusted HR was 2.11 (95% CI, 1.19 ~ 3.76) for Q4 versus Q1 of GDF-15 (*p* for trend =0.01), and was 1.23 (95% CI, 1.03 ~ 1.48) for each 1 SD increase in GDF-15 (data not shown). After adjusting for history of kidney stone (395 cases out of 4309 subjects), the adjusted HR was 2.40 (95% CI, 1.34 ~ 4.29) for Q4 versus Q1 of GDF-15 (*p* for trend < 0.01), and was 1.39 (95% CI, 1.16 ~ 1.65) for each 1 SD increase in GDF-15 (data not shown). Among 4244 individuals with baseline eGFR ≥60 mL/min/1.73 m^2^, 145 developed CKD. The adjusted HR was 2.08 (95% CI, 1.14 ~ 3.78; *p* for trend =0.02), and 1.22 (95% CI, 1.01 ~ 1.48) for Q4 versus Q1 and per 1 SD increase of GDF-15, respectively (data not shown). A total of 182 subjects developed AKI during the follow-up, and 13 out of them (7.14%) were later re-classified as CKD. After excluding subjects with AKI prior to CKD, the adjusted HR was 2.32 (95% CI, 1.25 ~ 4.30; *p* for trend < 0.01), and 1.33 (95% CI, 1.10 ~ 1.60) for Q4 versus Q1 and per 1 SD increase of GDF-15, respectively (data not shown).

### eGFR decline and incidence of eGFR-based CKD in relation to GDF-15

A total of 2744 had repeated eGFR values after a mean follow-up of 16.6 ± 1.49 years. The mean eGFR values at baseline and follow-up were 90.2 ± 12.8 and 66.2 ± 15.1 mL/min/1.73 m^2^, respectively (*N* = 2744). The association of eGFR change in relation to GDF-15, both in quartiles and per 1 SD increase, is presented in Table 3 (Analysis 2). As compared to Q1 of GDF-15, Q4 was associated with a greater eGFR decline during follow-up (− 2.42 mL/min/1.73 m^2^ (95% CI, − 3.91 ~ − 0.94); *p* for trend < 0.01) after multivariate adjustment including baseline eGFR. In addition, each 1 SD increase in GDF-15 was associated with a decline in eGFR of − 0.97 mL/min/1.73 m^2^ (95% CI, − 1.49 ~ − 0.45; *p* < 0.001) over the follow-up period. Results were consistent in participants with baseline eGFR ≥60 mL/min/1.73 m^2^ (*N* = 2713; B = -0.92; 95% CI, − 1.49 ~ − 0.36, *p* < 0.01) (data not shown).

**Table 3:** Change in estimated glomerular filtration rate in relation to baseline growth differentiation factor-15 (GDF-15)

	No. of subjects	B (95% CI) ^a^	*p*
GDF-15 Q1	790	Reference	–
GDF-15 Q2	763	−1.04 (−2.30, 0.22)	0.10
GDF-15 Q3	646	−1.67 (−3.01, −0.32)	0.02
GDF-15 Q4	545	−2.42 (− 3.91, − 0.94)	< 0.01
Per 1 standard deviation change in GDF-15	2744	− 0.97 (− 1.49, − 0.45)	< 0.001

*CI* confidence interval, *eGFR* estimated glomerular filtration rate according to the combined Chronic Kidney Disease Epidemiology Collaboration creatinine and cystatin C equation

^a^ Adjusted for waist circumference, smoking, high alcohol consumption, systolic blood pressure, low-density lipoprotein cholesterol, C-reactive protein level, diabetes, cardiovascular disease, anti-hypertensive drug medication, and baseline estimated glomerular filtration rate

Out of the 2713 individuals with baseline eGFR ≥60 mL/min/1.73 m^2^, 862 developed CKD, as defined by eGFR < 60 mL/min/1.73 m^2^. After adjusting for covariates including baseline eGFR, the odds ratio (OR) comparing Q4 vs Q1 of GDF-15 was 1.41 (95% CI, 1.06 ~ 1.89; *p* for trend =0.02) for developing eGFR-based CKD. The corresponding OR was 1.15 (95% CI, 1.04 ~ 1.28) per 1 SD increase in GDF-15 (Analysis 3: data not shown). Before re-examinations, 43 participants developed AKI based on ICD codes, of whom 26 (60.5%) were later identified to have eGFR-based CKD during re-examinations. After excluding them, the adjusted OR was 1.14 (95% CI, 1.03 ~ 1.27) for developing eGFR-based CKD per 1 SD increase in GDF-15.

## Discussion

Our findings suggest that GDF-15 is associated with increased incidence of CKD and eGFR decline. The association was independent of baseline eGFR, smoking, waist circumference, CRP, etc., and remained after controlling for competing risk of death. GDF-15, therefore, may be a useful marker of increased risk of CKD.

GDF-15 is a distant member of the transforming growth factor-β (TGF-β) family [25]. It has consistently been associated with deterioration of kidney function or adverse outcomes in patients with existing kidney diseases [9–12]. GDF-15 has also been investigated in relation to the development of CKD [7, 8], yet the association has remained uncertain. Carlsson, et al. used a discovery cohort (the PIVUS Study; *N* = 687, mean age = 70 years) and a replication cohort (the ULSAM study; *N* = 360, mean age = 78 years) to identify predictors of eGFR decline from 80 CVD biomarkers [8]. Whereas GDF-15 was initially observed to be associated with eGFR decline during a 5-year follow-up period, the association disappeared after adjusting for eGFR at baseline. The validity of this study may be affected by the relatively small sample size, the elderly population and potential survival bias. Results from the Framingham Offspring cohort study (*N* = 2614, mean follow-up period = 9.5 years) [7] suggested a superior predictive value of GDF-15 for incident CKD (estimated by one eGFR measurement). The association of GDF-15 with CKD was independent of baseline eGFR.

Even though the exact production rate and the kidney clearance rate is currently not available in our understanding of GDF-15, as a 24.5-kDa active circulating dimeric protein [26], levels of GDF-15 may largely depend upon renal excretory function for elimination. In support of this view, GDF-15 concentration negatively correlated with eGFR in the current and previous studies [8, 13], and was decreased by kidney function improvement through kidney transplantation [27]. Nevertheless, a strong association of GDF-15 with incident CKD was observed in our study and the study by Ho, et al. [7], even after adjustment for baseline eGFR, which may abolish some of the doubts about the kidney elimination dependence. Noteworthily, some potential confounders were not taken into account in the study by Ho, et al. [7]. We confirmed previous findings [14], suggesting that smoking, obesity, hs-CRP, etc. are strongly correlated with GDF-15 levels, and these are factors that also have been associated with CKD [28–30]. Our finding provides added evidence that GDF-15 was significantly associated with incidence of CKD and eGFR decline even after adjustments for baseline eGFR as well as other potential risk factors.

Dysfunction of the heart or kidney could potentially induce dysfunction of the other organ [1, 5, 6], known as a pathologic condition termed the cardiorenal syndrome [31]. In our sensitivity analysis, GDF-15 and NT-proBNP were mutually adjusted for each other in the multivariate model. GDF-15 but not NT-proBNP remained significantly associated with CKD and eGFR decline, suggesting that GDF-15 might be more specific to kidney outcomes than other CVD biomarkers. Moreover, the competing risk of death is usually high in studies on geriatric populations or with long-term follow-ups, and may largely bias the findings [32, 33]. We demonstrated for the first time that the fully-adjusted association of GDF-15 with eGFR decline and CKD remained after controlling for death as a competing risk.

The kidney can be an important source of GDF-15. In kidney tissue from adult rats, GDF-15 mRNA expression was mainly detected in the S3 segment of the nephron and the collecting ducts by in situ hybridization [34]. In response to stimuli such as surgery, toxin, ischemia, and hyperoxia, GDF-15 can be immediately induced in kidney, possibly through TNF- and p53-dependent and -independent pathways, and acts as a regulator of inflammation, cell survival, proliferation, and apoptosis [35]. Duong et al., observed that when stimulated by metabolic acidosis, GDF-15 expression was strongly induced in mouse kidney outer medullary collecting duct [36]. The increased GDF-15 played a key role in collecting duct lengthening by triggering compensatory proliferation of acid-secreting intercalated cells [36]. In contrast, genetic deletion of GDF-15 aggravated tubular and interstitial injury, which resulted in glycosuria and polyuria in mice with diabetes [37]. Meanwhile, epidemiological evidence showed that circulating levels of GDF-15 were closely correlated with mRNA expression of GDF-15 in renal tubulointerstitium, and significantly predicted risk of disease progression in patients with CKD [11]. Similarly, urinary GDF-15 was tightly linked with proximal tubule damage and kidney function decline in diabetic patients [38]. Based on the above-mentioned evidence, it is speculated that the observed association between plasma GDF-15 and incident CKD might at least partly be due to enhanced GDF-15 expression in kidney as a protective response against early-stage renal damage.

Strengths of this study included a prospective study design and a long-term follow-up. In addition, endpoints were retrieved from hospital registers with national coverage and a high case validity of the CKD diagnosis, as well as based on eGFR determined from a combination of plasma creatinine and cystatin C. There were several limitations that need to be mentioned. Our findings were descriptive in nature and provided limited information on mechanisms. Urine albumin is a strong predictor of incident CKD but data on albuminuria was not available. This did not affect eGFR calculation, but unfortunately we could not examine whether GDF-15 was associated with CKD independently of albuminuria. Moreover, residual confounding could result from other known risk factors, e.g. serum uric acid, family history of CKD, viral hepatitis, autoimmune disease and use of nephrotoxic agents as well as from unknown risk factors, even though extensive adjustments have already been made. Thus, further studies are still needed to further confirm the relationship.

CKD cases identified among hospital registers may not include less severe cases which were treated in primary care or were asymptomatic, and thus tended to underestimate CKD incidence. On the contrary, eGFR-based CKD may overestimate CKD incidence by using one eGFR measurement instead of repeated testing 3 months apart [21]. As a result, huge difference existed in different measures of CKD incidence, both of which might not be exactly the same as the true incidence. However, since GDF-15 was consistently associated with eGFR decline, incidence of register-based CKD as well as eGFR-based criteria, the overall results strongly supported a relationship with clinical CKD outcomes. Another important limitation is that the calculation of eGFR change may be influenced since the methods of measuring cystatin C and creatinine at baseline were different from those at the follow-up. Nevertheless, we expect that any bias introduced by these different measurements should be non-differential in relation to GDF-15 levels, and a greater decline in eGFR from baseline to follow-up could still reflect deteriorated kidney function. Therefore, we consider that the association between GDF-15 and eGFR decline was valid.

## Conclusions

In conclusion, this study indicated that elevated GDF-15 levels predicted incidence of CKD and eGFR decline in the general population, independent of a wide range of potential risk factors and competing risk of death.

## Data Availability

The data that support the findings of this study are available from Lund University, but restrictions apply to the availability of these data, which were used under license for the current study, and so are not publicly available. Data are however available from the authors upon reasonable request and with permission of Lund University.

## Abbreviations

AU: Arbitrary units
CI: Confidence interval
CKD: Chronic kidney disease
CRP: C-reactive protein
eGFR: Estimated glomerular filtration rate
ESRD: End-stage renal disease
GDF-15: Growth differentiation factor 15
HR: Hazard ratio
IDMS: Isotope-dilution mass spectrometry-traceable
LDL: Low density lipoprotein
MDC-CV: Malmö Diet and Cancer Cardiovascular
NT-proBNP: N-terminal pro-B-type natriuretic peptide
OR: Odds ratio
SD: Standard deviation
TGF-β: Transforming growth factor-β
